# Challenges in Balancing Hemostasis and Thrombosis in Therapy Tailoring for Hemophilia: A Narrative Review

**DOI:** 10.3390/ijms27031373

**Published:** 2026-01-29

**Authors:** Gili Kenet, Sarina Levy-Mendelovich, Tami Livnat, Benjamin Brenner

**Affiliations:** 1 National Hemophilia Center and Coagulation Institute, Sheba Medical Center, Ramat Gan 52621, Israeltami.livnat@sheba.health.gov.il (T.L.); 2 Amalia Biron Research Institute of Thrombosis and Hemostasis, Faculty of Medicine, Tel Aviv University, Tel Aviv 6997801, Israel; 3 Talpiot Medical Leadership Program, Sheba Medical Center, Tel Hashomer 52621, Israel; 4 Department of Hematology and Bone Marrow Transplantation, Rambam Health Care Campus, Haifa 3109601, Israel; 5 The Ruth and Bruce Rappaport Faculty of Medicine, Technion, Haifa 3200003, Israel

**Keywords:** Hemophilia, thrombosis, rebalancing agents

## Abstract

Hemostasis and thrombosis reflect a delicate balance, regulated by the interplay between procoagulant and anticoagulant mechanisms. Hemophilia is traditionally viewed as a bleeding disorder, but emerging evidence highlights the paradoxical risks of thrombosis in hemophilia patients. We explore the landscape of hemophilia management, emphasizing challenges of balancing hemostasis in the context of aging, novel non-factor replacement therapies (NRTs), and comorbidity-driven thrombotic complications. Therapeutic approaches, including innovative NRTs, such as emicizumab, or rebalancing agents (e.g., concizumab, marstacimab, fitusiran), offer promising advancements in bleeding prophylaxis but may increase thrombotic risks. Conversely, novel anticoagulants, such as FXI inhibitors, offer potential thrombosis protection with minimal bleeding risk. Our review examines the impact of aging-related comorbidities, including cardiovascular disease, atrial fibrillation, HIV-associated complications, and acute coronary syndromes, on thrombotic risk in hemophilia patients. Evidence-based strategies for balancing hemostasis and thrombosis are outlined alongside experimental models, thrombin generation assays, and advancements in rebalancing coagulation through natural anticoagulant modulation. FXI inhibition emerges as a paradigm shift in thrombosis management, offering reduced bleeding risks while preserving vascular health. Finally, this review highlights the need for global laboratory assays to personalize treatments, emphasizing strategies to optimize safety and efficacy, particularly as hemophilia patients live longer with complex comorbidity profiles.

## 1. Introduction

Hemostasis is a tightly regulated process involving the coordinated interaction of platelets, coagulation proteins, and vascular endothelium to maintain vascular integrity, minimize blood loss, and prevent pathological clot formation [1]. When vascular injury occurs, primary hemostasis is initiated with platelet adhesion and aggregation at the site of damage, forming a temporary plug. Secondary hemostasis stabilizes the plug by converting fibrinogen to insoluble fibrin via thrombin-mediated activation, leading to clot formation. Thrombin, the central enzyme in coagulation, drives fibrin deposition and amplifies the activation of coagulation factors and platelets, solidifying the clot [2].

Thrombin generation is composed of the initiation phase, mediated by tissue factor (TF)-bearing cells, that produces small quantities of FXa and thrombin, and the amplification phase that generates a robust “thrombin burst” on the surface of activated platelets [3]. This process relies on macromolecular complexes, extrinsic tenase (FVIIa:TF), intrinsic tenase (FIXa:FVIIIa), and prothrombinase (FXa:FVa), assembled on procoagulant phospholipid surfaces. In physiological conditions, this response is localized, ensuring effective clot formation without inducing thrombosis. Anticoagulant systems, mediated by circulating inhibitors such as tissue factor pathway inhibitor (TFPI) and antithrombin (AT), as well as endothelial-localized regulators like activated Protein C (APC), tightly control thrombin generation [2].

A critical factor in this hemostatic balance is vitamin K-dependent carboxylation, which is essential for the production of several coagulation factors (II, VII, IX, and X) and natural anticoagulant proteins (Protein C and Protein S). Vitamin K serves as a cofactor for the post-translational modification of glutamic acid residues into γ-carboxyglutamic acid, allowing these proteins to bind calcium and phospholipid surfaces at sites of injury [4]. Deficiency in vitamin K impairs both procoagulant and anticoagulant protein function, increasing the risk of bleeding or thrombosis [4,5].

Warfarin, a widely used anticoagulant, inhibits vitamin K epoxide reductase (VKORC1), preventing vitamin K recycling into its active form. This interruption reduces carboxylation and impairs the functionality of both procoagulant and anticoagulant proteins, providing effective anticoagulation but requiring careful dose management due to the risk of bleeding [6].

Advances in the understanding of thrombin generation have revolutionized the management of severe bleeding disorders, like hemophilia, moving beyond replacement therapy. These insights underscore the importance of balancing procoagulant and anticoagulant forces in achieving hemostatic regulation [2].

The intricate interplay between thrombosis and bleeding is evident in both physiological and pathological hemostasis. This review will examine hemostatic therapies in the context of hemophilia, an inherited coagulopathy. We will address hemophilia as a well-characterized and extensively studied clinical model; however, it is not a surrogate for all rare bleeding disorders. This review will have a particular focus on aging and novel non-replacement therapies. We will also briefly discuss novel anticoagulant drugs and their therapeutic implications for hemostatic regulation, as well as promising murine models based upon rebalancing coagulation.

## 2. Thrombosis in Hemophilia: The Emerging Challenge of Aging and Comorbidities

**Figure 1:**
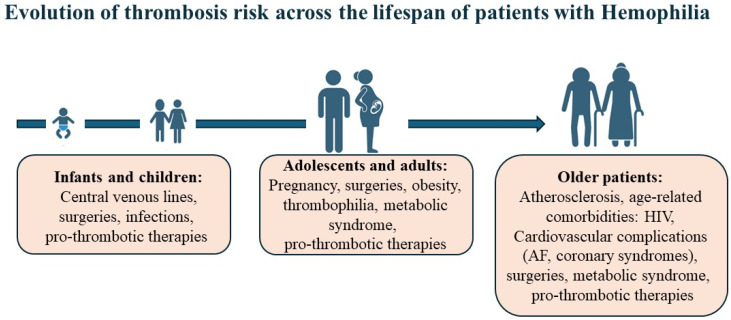
Evolution of thrombotic risks across the life span of patients with hemophilia. In children, adolescents, and adults with rare bleeding disorders, thrombosis is almost always associated with coexisting risk factors such as presence of central venous lines, surgeries, inherited thrombophilia, or metabolic syndromes and may be exacerbated by potentially pro-thrombotic therapies. In elderly patients, however, the low but notable risk of thrombotic events increases with the abundance of cardiovascular complications and age-related comorbidities. In selected cases and high-risk situations, anticoagulant therapy or prophylaxis may be considered along with hemostatic care. AF-Atrial fibrillation; HIV-Human immune deficiency virus.

Hemophilia A (HA) and B (HB) are X-linked inherited bleeding disorders caused by defective synthesis of coagulation factors VIII or IX, resulting in impaired thrombin generation [7]. While traditionally regarded as disorders of bleeding, growing evidence reveals a paradoxical risk of thrombotic complications in these patients, driven by comorbidities and the thrombogenic effects of modern hemostatic therapies, underscoring the intricate interplay between bleeding and clotting in this complex patient population. Advances in treatment have significantly improved life expectancy for people with hemophilia, aligning it with the general male population [8]. This shift, however, has introduced new challenges, including age-related comorbidities such as cardiovascular events, HIV-associated complications, and atrial fibrillation, challenging the long-held assumption that hemophilia’s hypocoagulable state protects against thrombotic cardiovascular disease [9,10]. While thrombotic complications remain rare in patients with hemophilia and other rare bleeding disorders, the risk of such events has been observed to increase with advancing age. Figure 1 summarizes the risk for thrombotic complications across the life span of patients with hemophilia. In children, adolescents, and adults with rare bleeding disorders, thrombosis is almost always associated with coexisting risk factors such as presence of central venous lines, inherited thrombophilia, or metabolic disorders and may be exacerbated by potentially pro-thrombotic therapies (Table 1). In older patients, however, the low but notable risk of thrombotic events increases with the abundance of cardiovascular complications and age-related comorbidities (Figure 1).

The hallmark of hemophilia care has traditionally been prophylaxis with replacement products. For patients with inhibitors, bypassing agents (BPAs) are widely used to restore hemostasis. BPAs, such as recombinant activated factor VII (rFVIIa, NovoSeven^®^, Novo Nordisk, Bagsvaerd, Denmark) and activated prothrombin complex concentrate (aPCC, FEIBA^®^ Takeda, Vienna, Austria), have a vital role in managing bleeding in patients with inhibitors by bypassing the need for factor VIII or IX to generate thrombin. Clinical trials have demonstrated their efficacy in both acute and prophylactic settings, offering a reliable option for inhibitor patients. However, BPA use has been linked to an increased risk of thrombotic complications, particularly in older patients. Studies suggest that careful monitoring is needed to mitigate thrombotic risks, particularly when used concomitantly with other prothrombotic agents or in patients with underlying cardiovascular disease [7,11].

Before the implementation of pathogen inactivation techniques in the 1980s, plasma-derived replacement products often transmitted HIV and hepatitis C infections to individuals with hemophilia. In patients with HIV, chronic inflammation, immune activation, and antiretroviral therapy (ART)-induced metabolic changes significantly contribute to heightened cardiovascular and metabolic risks [12,13,14]. HIV independently increases atherosclerotic cardiovascular disease (ASCVD) risk through persistent immune activation, endothelial dysfunction, and dysregulated lipid and glucose metabolism, even with ART-mediated viral suppression.

The American Heart Association identifies HIV-associated inflammation and immune activation as major drivers of endothelial dysfunction and atherogenesis, while certain ART regimens, particularly protease inhibitors, can exacerbate dyslipidemia, insulin resistance, and fat redistribution. The cardiovascular risk posed by HIV is comparable to traditional factors like diabetes or hypertension. Furthermore, certain ART agents commonly cause lipid abnormalities such as hypertriglyceridemia, compounding cardiovascular risk. Increased rates of myocardial infarction and advanced atherosclerosis are particularly associated with HIV and low CD4 T-cell counts [15].

These findings underscore the need for tailored strategies to address the cumulative impact of HIV and hemophilia on cardiovascular health. ART selection should minimize metabolic complications, with regular monitoring of atherosclerotic cardiovascular disease (ASCVD) risk, lipid profiles, and glucose metabolism recommended. Statin therapy is advised for primary prevention in patients with HIV at elevated cardiovascular risk, with careful management of potential interactions between statins and ART [15].

Atrial fibrillation (AF) and acute cardiovascular events represent overlapping challenges in patients with hemophilia, particularly as they age and develop comorbidities. Both scenarios demand careful coordination between cardiology and hematology teams, with strategies tailored to balance thrombotic risk against the inherent bleeding propensity of hemophilia.

With the advancing age of patients with hemophilia, atrial fibrillation (AF) becomes an increasingly relevant clinical challenge, particularly regarding the balance between anticoagulation for stroke prevention and the elevated bleeding risk inherent to hemophilia [16]. Due to the lack of consensus in the literature, management requires a cautious, individualized approach [17]. Estimating stroke risk with the CHA2DS2-VASc score, along with hemophilia severity, is pivotal for guiding therapeutic decisions. For moderate or severe hemophilia, clotting factor replacement is critical when initiating anticoagulation or dual antiplatelet therapy (DAPT), with trough factor VIII or IX levels maintained at 20–30 IU/dL for routine prophylaxis and dual antiplatelet therapy (DAPT), and ≥50 IU/dL during invasive procedures or major bleeding episodes [18,19].

Catheter ablation and left atrial appendage occlusion (LAAO) are effective alternatives to long-term anticoagulation for stroke prevention in patients with contraindications. However, LAAO necessitates short-term anticoagulation post procedure, followed by DAPT and lifelong aspirin, which may still present bleeding risks and require close hematologic oversight [17,20]. These strategies underscore the importance of individualized, multidisciplinary care to balance thrombotic and bleeding risks in hemophilia patients with AF.

Building upon the considerations for anticoagulation in AF, the acute management of cardiac events further underscores the critical need for individualized approaches, early clotting factor replacement, and multidisciplinary planning to optimize patient outcomes. Individualized risk assessment should account for hemophilia severity, bleeding history, presence of inhibitors, and comorbidities such as HIV or hepatic dysfunction [21]. Clotting factor replacement should be administered promptly prior to initiating antithrombotic therapy or invasive procedures, targeting factor VIII or IX levels of 80–100%, with adjustments based on inhibitors and individual pharmacokinetics [22,23,24].

Management of acute coronary syndrome (ACS) typically includes antiplatelet agents (aspirin, P2Y12 inhibitors) and anticoagulants (unfractionated heparin or bivalirudin). Agents with short half-lives or reversibility are preferred to facilitate rapid cessation in the event of bleeding complications [25]. Warfarin is generally avoided due to unpredictable bleeding risks, while the duration and intensity of DAPT should be carefully minimized. Direct oral anticoagulants (DOACs) may be considered in select cases, though data on their safety and efficacy in hemophilia remain limited [26,27].

For invasive procedures, transradial cardiac catheterization is preferred to reduce access-site bleeding. Bare-metal stents or drug-coated balloons may be favored over drug-eluting stents to shorten DAPT duration, which is especially important for patients at high bleeding risk [28]. These tailored approaches highlight the importance of balancing thrombotic management with bleeding prevention in this complex patient population.

## 3. Novel Therapies: Emerging Complications of Thrombosis and Thrombotic Microangiopathy

**Figure 2:**
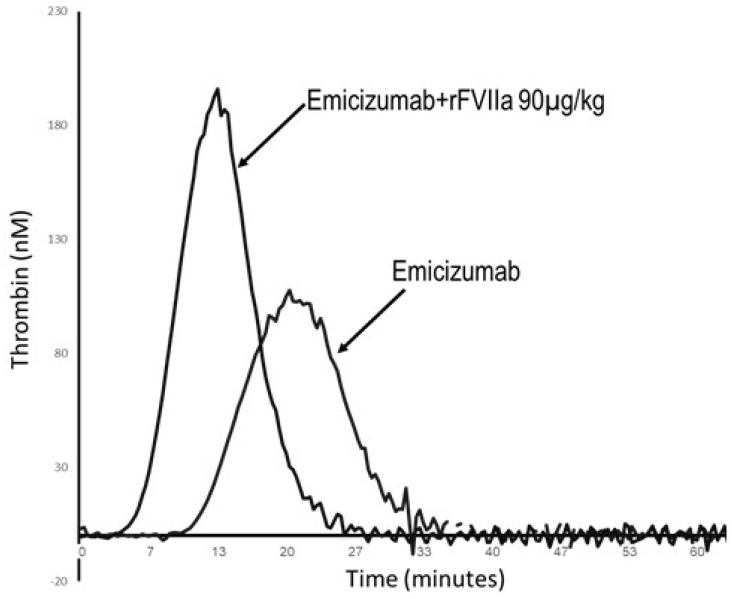
Thrombin generation profile in hemophilia patient treated with novel non-factor agents. This figure shows the thrombin generation curve of a hemophilia A patient with inhibitor on emicizumab with and without rFVIIa (90 µg/kg), applied peri-operatively [29]. Combination therapy of a prophylactically applied emicizumab along with recombinant FVIIa enhances thrombin generation, underscoring the importance of thrombin generation-based monitoring to mitigate thrombotic risks.

The treatment landscape for hemophilia has recently evolved beyond conventional replacement and bypassing therapies, entering a new era defined by non-factor agents that aim to provide consistent hemostatic correction. Novel non-replacement therapies (NRTs), such as emicizumab for HA, and emerging rebalancing agents that enhance thrombin generation via targeted regulatory pathways offer promising alternatives for the treatment of hemophilia and potentially other rare bleeding disorders. While these newer therapies provide consistent prophylaxis and lower treatment burden, their potential thrombotic risks warrant careful consideration and vigilance. For specification of therapy-related complications, see Table 1. Figure 2 shows that combination therapies (such as emicizumab and bypass agents) may further increase pro-thrombotic potential, underscoring the importance of thrombin generation-based monitoring to mitigate thrombotic risk. It should be noted that some of the novel non-replacement hemophilia medications have a long half-life and their complete clearance may take days to months [7].

Emicizumab, the monoclonal human bispecific antibody targeting FIX and FX and mimicking FVIII activity, was found to be an effective subcutaneous therapy for patients with HA at all ages [30]. Its safety was described among all age groups, including elderly patients with cardiovascular risk factors [31].

Thrombotic events, including thrombotic microangiopathy (TMA), have been reported with the use of emicizumab, particularly when combined with bypassing agents such as aPCC [11,32]. Data on these adverse events suggest that caution is needed, especially at higher doses of aPCC.

Managing such thrombotic complications warrants early recognition of symptoms such as thrombocytopenia, hemolysis, or renal impairment, followed by discontinuation of the implicated therapy. In more severe cases, anticoagulation may be required, although careful consideration of the bleeding risk is necessary. Supportive care, including monitoring renal function and vascular health, should be part of the management plan for high-risk patients. Regular assessment using biomarkers like lactate dehydrogenase (LDH) or D-dimer may assist in detecting early signs of TMA or thrombotic events in patients on non-factor therapies [32].

The advent of novel therapies for hemophilia is shifting the focus toward restoring hemostatic balance by targeting natural anticoagulant mechanisms, including protein C/S, tissue factor pathway inhibitor (TFPI), and antithrombin (AT).

These treatments seek to restore the hemostatic balance from the opposite direction: by reducing endogenous anticoagulant activity rather than supplementing missing clotting factors. These agents hold promise for sustained, simplified management of HA and HB. Furthermore, they offer a viable therapeutic option for patients who develop inhibitors following replacement therapy, addressing an unmet clinical need [33].

A critical step in the development of these therapies has relied on rigorous preclinical evaluations in murine models, which remain the cornerstone of hemophilia research. Factor VIII-deficient knockout (F8-KO) and factor IX-deficient knockout (F9-KO) mice remain the gold standards for modeling hemophilia A and B, respectively, due to their genetic tractability and ability to replicate severe bleeding phenotypes [34]. These models have profoundly influenced the study and refinement of rebalancing agents, facilitating the translation of experimental therapies into clinical applications.

One example is ALN-AT3, Fitusiran, a subcutaneous RNA interference (RNAi) therapeutic that targets AT. Preclinical studies in HA mice demonstrated robust, dose-dependent, and durable reductions in AT levels [35]. Subsequent clinical trials confirmed its bleeding prophylaxis efficacy in HA and HB patients, with and without inhibitors. Nonetheless, FDA drug approval has been delayed, as with the original dose regimen (80 mg QM) thromboembolic complications have been reported in several participants of the phase 2 and 3 studies. Initial risk mitigation for thrombosis followed and included screening and exclusion of participants with thrombophilia or certain thrombotic risk factors. Furthermore, antithrombin levels were evaluated as a potential modifiable target for risk mitigation, and a revised AT-based dosing regimen has been implemented, targeting AT activity levels of 15–35%, and escalating or de-escalating dose/frequency based on individual participant response, as determined by measurements of AT activity levels [36,37]. Qfitlia (fitusiran) received FDA approval on March 28, 2025, for routine prophylaxis to prevent or reduce the frequency of bleeding episodes in adults and pediatric patients aged 12 years and older, with or without factor VIII or IX inhibitor [38].

Other promising rebalancing agents are concizumab and marstacimab, which selectively bind to the K2 domain of the tissue factor pathway inhibitor (TFPI), thus restoring thrombin generation [39]. TFPI plays a pivotal role in regulating thrombin generation by inhibiting the extrinsic tenase and prothrombinase complexes. In hemophilia, where intrinsic pathway activity is impaired due to a deficiency in factor VIII or IX, TFPI exacerbates the bleeding phenotype through its inhibitory effects on the extrinsic coagulation pathway. Genetically and pharmacologically reducing TFPI activity, particularly hematopoietic or platelet-derived TFPI, has shown significant efficacy in mitigating bleeding and enhancing fibrin formation in murine hemophilia models. These findings set the foundation for therapeutic development. Notably, some anti-TFPI studies were prematurely terminated due to the occurrence of venous and arterial thromboses [40]. The FDA approved Alhemo (concizumab) on 20 December 2024. This approval was significant as it marked the first time a subcutaneous prophylactic therapy was available for patients with HA or HB with inhibitors. Marstacimab (Hympazi) was approved in October 2025 for adults/adolescents with HA/HB [41,42,43].

Rebalancing agents targeting protein C and protein S have also gained traction as promising approaches for hemophilia management [44,45,46]. Early studies by Magisetty J. et al. [44] and Jiang M. et al. [44] demonstrated that selective inhibition of the anticoagulant activity of activated protein C (APC), while preserving its cytoprotective signaling, significantly reduced hemophilic arthropathy severity in factor VIII-deficient mice. These promising results are paving the way for clinical trials exploring protein C pathway modulation as a new avenue for hemophilia treatment.

The development of rebalancing agents has transformed the therapeutic landscape for hemophilia, particularly for patients with limited or no alternative options. By addressing the upstream regulation of hemostasis, these agents offer innovative strategies for improving outcomes and quality of life in patients with RBD [47].

This concept is further illustrated by representative thrombin generation (TG) assays obtained from patients treated with novel therapies, underscoring the complexity of maintaining hemostatic balance in clinical practice (Figure 2). Representative thrombin generation profiles illustrate ex vivo plasma assays from two inhibitor patients treated with novel non-factor agents. Panel A depicts a patient with severe HA and a high-titer FVIII inhibitor who received emicizumab prophylaxis and experienced a breakthrough bleed managed with rFVIIa (90 µg/kg). Panel B shows a patient with HB and inhibitor treated with Qfitlia (fitusiran) who similarly required rFVIIa (45 µg/kg) following a bleeding episode. Improvement of TG is noted, yet during breakthrough bleeding episodes the use of bypass agents may yield normal or supra-physiologic TG. Thus, caution is required when breakthrough bleeding episodes are treated under NRT prophylaxis. These findings highlight the unmet need for global laboratory assays capable of monitoring overall hemostatic potential to prevent thrombotic complications. Global assays such as the TG assay may provide valuable tools for therapy tailoring and assess potential synergistic effects between agents in order to mitigate potential pro-thrombotic risks.

The principle of hemostatic rebalancing can operate bidirectionally: just as bleeding disorders may be mitigated by procoagulant interventions, a prothrombotic state can be alleviated by introducing a compensatory bleeding phenotype. This concept is exemplified in murine models of severe protein C deficiency (SPCD), a condition otherwise incompatible with life due to disseminated embryonic thrombosis and early lethality [48]. In these models, survival was achieved by introducing a concomitant F8 gene knockout, thereby generating an HA phenotype that effectively counterbalanced the hypercoagulable state. This innovative genetic strategy not only enabled the establishment of a viable SPCD model but also provided proof of concept for hemostatic rebalancing as a therapeutic approach. Moreover, it opened the way for successful gene therapy rescue of the PC pathway, underscoring the translational potential of rebalancing mechanisms in managing thrombophilic disorders [49].

## 4. Novel Anticoagulant Drug Targets: Exploring the Role of FXI in Rebalancing Hemostasis

The concept of rebalancing hemostasis extends beyond the treatment of bleeding disorders and into the realm of novel anticoagulant therapies. Factor XI (FXI) has recently emerged as a promising drug target due to its unique role in thrombus formation. FXI deficiency is associated with a reduced risk of thromboembolic disease, yet patients with naturally occurring FXI deficiency, such as those with FXI levels below 15%, rarely develop spontaneous bleeding [50]. This phenotype underscores the potential of FXI inhibition as a therapeutic strategy to lower thrombotic risk without posing the high bleeding risk typically associated with other anticoagulants [51].

FXI inhibition represents a novel pharmacological avenue for rebalancing hemostasis in conditions of excessive thrombosis. By selectively targeting FXI, these new agents modulate the coagulation cascade at a point where thrombin generation is dampened without interfering significantly with primary hemostasis. Thus, FXI-targeted therapies provide a unique mechanism to achieve a balanced anticoagulant effect, reducing the pathological thrombotic response while preserving baseline clotting capacity essential for vascular integrity [52].

In phase 2 studies, drugs that inhibit FXI or FXIa prevent venous thromboembolism after total knee arthroplasty as well as, or better than, low molecular weight heparin. Patients with heart disease on FXI or FXIa inhibitors experienced less bleeding than patients taking DOACs. Based on these early results, phase 3 trials have been initiated that compare drugs targeting FXI and FXIa to standard treatments or placebo [53].

This approach aligns conceptually with the rebalancing principles applied to bleeding disorders, where procoagulant forces are restored by inhibiting natural anticoagulant mechanisms. In FXI-targeted anticoagulation, the inverse principle is applied: anticoagulant effects are achieved in a manner that avoids tipping the balance toward pathological bleeding. The ability of FXI-targeted therapies to selectively modulate hemostasis while minimizing bleeding risk offers valuable insights into extending rebalancing strategies toward thrombosis management, further bridging the concepts of bleeding and clotting regulation [51].

## 5. Summary and Future Perspectives

The intricate interplay between hemostasis and thrombosis presents unique challenges in managing bleeding disorders, particularly as therapeutic advancements introduce novel complexities. This review underscores the evolving landscape of hemophilia treatment, highlighting the paradoxical risks posed by aging, modern therapies, and comorbid conditions. From thrombotic complications in older hemophilia patients to the advent of rebalancing agents and non-factor therapies, the balance between bleeding control and thrombotic risk continues to demand individualized, multidisciplinary care. Recent innovations, including protein C and TFPI-targeting therapies, emphasize the growing reliance on precision medicine to optimize treatment outcomes [54]. Similarly, emerging anticoagulant strategies, such as FXI inhibition, offer promise in further bridging the regulation of hemostasis and thrombosis, paving the way for safer, tailored interventions.

Looking ahead, the development of murine models for severe bleeding and thrombophilic disorders remains integral to advancing this field, offering proof of concept and preclinical validation for novel therapies. Innovations such as gene therapy and RNA interference strategies demonstrate the potential to address unmet needs while enhancing quality of life and survival for patients with hemophilia and rare bleeding disorders. The therapeutic principle of hemostatic rebalancing, whether applied to mitigate bleeding or thrombosis, exemplifies the cross-disciplinary progress in understanding and managing the complex biology of coagulation. Continued research into individualized risk assessment, advanced imaging, biomarker screening, and tailored dosing regimens promises a future of safer, more effective treatment paradigms for the dual challenges of hemostasis and thrombosis.

In conclusion, this review underscores the importance of multidisciplinary care, individualized risk assessment, and tailored therapeutic pathways in navigating the challenges of hemostasis and thrombosis. Advances in risk stratification, preclinical modeling, and gene therapy herald a future defined by precision medicine and safer, more effective management strategies for bleeding and thrombotic disorders.

## Figures and Tables

**Table 1:** Therapy-related thrombotic complications in patients with hemophilia.

Therapy/Agent	Mechanism	Potential Risk	Implications
Bypass agents	Thrombosis, DIC	Excess factor activation	Monitor thrombotic markers, use with caution in elderly inhibitor patients with cardiovascular risk factors
Emicizumab	Thrombosis, TMA	Overactivation of thrombin generation	Requires vigilance with aPCC co-administration
Rebalancing agents	Potential off-target effects	Alteration of coagulation balance	Limited real-world clinical experience, thrombosis reported in/after clinical trials, caution is advised in patients with risk factors

DIC—Disseminated intravascular coagulation, TMA—Thrombotic microangiopathy, aPCC—Activated Prothrombin Complex Concentrate.

## Data Availability

Upon request from the corresponding author.

## References

[1] Mannucci P.M., Franchini M. (2010). Mechanism of hemostasis defects and management of bleeding in patients with acute coronary syndromes. Eur. J. Intern. Med..

[2] Vu H.H., McCarty O.J.T., Favaloro E.J. (2024). Contact Activation: Where Thrombosis and Hemostasis Meet on a Foreign Surface, Plus a Mini-editorial Compilation (“Part XVI”). Semin. Thromb. Hemost..

[3] Wada H., Shiraki K., Shimpo H., Miyata T. (2025). Evaluation of Deficiency and Excessive Condition of Thrombin Burst Using Laboratory Tests. Thromb. Haemost..

[4] Brenner B., Sánchez-Vega B., Wu S.M., Lanir N., Stafford D.W., Solera J. (1998). A missense mutation in gamma-glutamyl carboxylase gene causes combined deficiency of all vitamin K-dependent blood coagulation factors. Blood.

[5] Hao Z., Jin D.Y., Stafford D.W., Tie J.K. (2020). Vitamin K-dependent carboxylation of coagulation factors: Insights from a cell-based functional study. Haematologica.

[6] Oldenburg J., Watzka M., Rost S., Müller C.R. (2007). VKORC1: Molecular target of coumarins. J. Thromb. Haemost..

[7] Chowdary P., Carcao M., Kenet G., Pipe S.W. (2025). Haemophilia. Lancet.

[8] Shapiro S., Benson G., Evans G., Harrison C., Mangles S., Makris M. (2022). Cardiovascular disease in hereditary haemophilia: The challenges of longevity. Br. J. Haematol..

[9] Soucie J.M., Nuss R., Evatt B., Abdelhak A., Cowan L., Hill H., Kolakoski M., Wilber N. (2000). Mortality among males with hemophilia: Relations with source of medical care. The Hemophilia Surveillance System Project Investigators. Blood.

[10] Makris M., Lassila R., Kennedy M. (2024). Challenges in ageing persons with haemophilia. Haemophilia.

[11] Li T., Huang D., Jiang Y. (2025). A real-world pharmacovigilance analysis of the FDA adverse event reporting system database for emicizumab. Thromb. Res..

[12] Feinstein M.J., Hsue P.Y., Benjamin L.A., Bloomfield G.S., Currier J.S., Freiberg M.S., Grinspoon S.K., Levin J., Longenecker C.T., Post W.S. (2019). Characteristics, Prevention, and Management of Cardiovascular Disease in People Living With HIV: A Scientific Statement from the American Heart Association. Circulation.

[13] Perkins M.V., Joseph S.B., Dittmer D.P., Mackman N. (2023). Cardiovascular Disease and Thrombosis in HIV Infection. Arterioscler. Thromb. Vasc. Biol..

[14] Wei S., Evans P.C., Strijdom H., Xu S. (2025). HIV infection, antiretroviral therapy and vascular dysfunction: Effects, mechanisms and treatments. Pharmacol. Res..

[15] Horberg M., Thompson M., Agwu A., Colasanti J., Haddad M., Jain M., McComsey G., Radix A., Rakhmanina N., Short W.R. (2024). Primary Care Guidance for Providers of Care for Persons with Human Immunodeficiency Virus: 2024 Update by the HIV Medicine Association of the Infectious Diseases Society of America. Clin. Infect. Dis..

[16] Philipp C. (2010). The aging patient with hemophilia: Complications, comorbidities, and management issues. Hematol. Am. Soc. Hematol. Educ. Program.

[17] Badescu M.C., Badulescu O.V., Butnariu L.I., Floria M., Ciocoiu M., Costache I.I., Popescu D., Bratoiu I., Buliga-Finis O.N., Rezus C. (2022). Current Therapeutic Approach to Atrial Fibrillation in Patients with Congenital Hemophilia. J. Pers. Med..

[18] Franchini M., Focosi D., Mannucci P.M. (2023). How we manage cardiovascular disease in patients with hemophilia. Haematologica.

[19] Atar D., Vandenbriele C., Agewall S., Gigante B., Goette A., Gorog D.A., Holme P.A., Krychtiuk K.A., Rocca B., Siller-Matula J.M. (2025). Management of patients with congenital bleeding disorders and cardiac indications for antithrombotic therapy. Eur. Heart J. Cardiovasc. Pharmacother..

[20] Joglar J.A., Chung M.K., Armbruster A.L., Benjamin E.J., Chyou J.Y., Cronin E.M., Deswal A., Eckhardt L.L., Goldberger Z.D., Gopinathannair R. (2024). and Peer Review Committee Members. 2023 ACC/AHA/ACCP/HRS Guideline for the Diagnosis and Management of Atrial Fibrillation: A Report of the American College of Cardiology/American Heart Association Joint Committee on Clinical Practice Guidelines. Circulation.

[21] Mannucci P.M. (2012). Management of antithrombotic therapy for acute coronary syndromes and atrial fibrillation in patients with hemophilia. Expert. Opin. Pharmacother..

[22] Chen H., Yang S. (2023). Acute coronary syndrome management in hemophiliacs: How to maintain balance?: A review. Medicine.

[23] Alblaihed L., Dubbs S.B., Koyfman A., Long B. (2022). High risk and low prevalence diseases: Hemophilia emergencies. Am. J. Emerg. Med..

[24] Boehnel C., Rickli H., Graf L., Maeder M.T. (2018). Coronary angiography with or without percutaneous coronary intervention in patients with hemophilia-Systematic review. Catheter. Cardiovasc. Interv..

[25] Franchini M., Tagliaferri A., Mannucci P.M. (2007). The management of hemophilia in elderly patients. Clin. Interv. Aging.

[26] Badulescu O.V., Scripcariu D.V., Badescu M.C., Ciocoiu M., Vladeanu M.C., Plesoianu C.E., Bojan A., Iliescu-Halitchi D., Tudor R., Huzum B. (2024). Debates Surrounding the Use of Antithrombotic Therapy in Hemophilic Patients with Cardiovascular Disease: Best Strategies to Minimize Severe Bleeding Risk. Int. J. Mol. Sci..

[27] Escobar M., Lassila R., Bekdache C., Owaidah T., Sholzberg M. (2025). Use of antithrombotic therapy in patients with hemophilia: A selected synopsis of the European Hematology Association—International Society on Thrombosis and Haemostasis—European Association for Hemophilia and Allied Disorders—European Stroke Organization Clinical Practice Guidance document. J. Thromb. Haemost..

[28] Vadalà G., Mingoia G., Astuti G., Madaudo C., Sucato V., Adorno D., D’Agostino A., Novo G., Corrado E., Galassi A.R. (2025). Coronary Revascularization in Patients with Hemophilia and Acute Coronary Syndrome: Case Report and Brief Literature Review. J. Clin. Med..

[29] Cohen O., Levy-Mendelovich S., Budnik I., Ludan N., Lyskov S.K., Livnat T., Avishai E., Efros O., Lubetsky A., Lalezari S. (2023). Management of surgery in persons with hemophilia A receiving emicizumab prophylaxis: Data from a national hemophilia treatment center. Res. Pract. Thromb. Haemost..

[30] Callaghan M.U., Negrier C., Paz-Priel I., Chang T., Chebon S., Lehle M., Mahlangu J., Young G., Kruse-Jarres R., Mancuso M.E. (2021). Long-term outcomes with emicizumab prophylaxis for hemophilia A with or without FVIII inhibitors from the HAVEN 1-4 studies. Blood.

[31] Misgav M., Brutman-Barazani T., Budnik I., Avishai E., Schapiro J., Bashari D., Barg A.A., Lubetsky A., Livnat T., Kenet G. (2021). Emicizumab prophylaxis in haemophilia patients older than 50 years with cardiovascular risk factors: Real-world data. Haemophilia.

[32] Abbattista M., Ciavarella A., Noone D., Peyvandi F. (2023). Hemorrhagic and thrombotic adverse events associated with emicizumab and extended half-life factor VIII replacement drugs: EudraVigilance data of 2021. J. Thromb. Haemost..

[33] Van Thillo Q., Hermans C. (2025). Rebalancing agents in hemophilia: Knowns, unknowns, and uncertainties. Haematologica.

[34] Sabatino D.E., Nichols T.C., Merricks E., Bellinger D.A., Herzog R.W., Monahan P.E. (2012). Animal models of hemophilia. Prog. Mol. Biol. Transl. Sci..

[35] Sehgal A., Barros S., Ivanciu L., Cooley B., Qin J., Racie T., Hettinger J., Carioto M., Jiang Y., Brodsky J. (2015). An RNAi therapeutic targeting antithrombin to rebalance the coagulation system and promote hemostasis in hemophilia. Nat. Med..

[36] Kenet G., Nolan B., Zulfikar B., Antmen B., Kampmann P., Matsushita T., You C.W., Vilchevska K., Bagot C.N., Sharif A. (2024). Fitusiran prophylaxis in people with hemophilia A or B who switched from prior BPA/CFC prophylaxis: The ATLAS-PPX trial. Blood.

[37] Young G., Kavakli K., Klamroth R., Matsushita T., Peyvandi F., Pipe S.W., Rangarajan S., Shen M.C., Srivastava A., Sun J. (2025). Safety and efficacy of a fitusiran antithrombin-based dose regimen in people with hemophilia A or B: The ATLAS-OLE study. Blood.

[38] Lu D., Dou F., Gao J. (2025). Fitusiran: The first approved siRNA therapy for hemophilia via reducing plasma antithrombin levels. Drug Discov. Ther..

[39] Chowdary P. (2020). Anti-tissue factor pathway inhibitor (TFPI) therapy: A novel approach to the treatment of haemophilia. Int. J. Hematol..

[40] Mahlangu J.N. (2021). Progress in the Development of Anti-tissue Factor Pathway Inhibitors for Haemophilia Management. Front. Med..

[41] Matsushita T., Shapiro A., Abraham A., Angchaisuksiri P., Castaman G., Cepo K., d’Oiron R., Frei-Jones M., Goh A.S., Haaning J. (2023). Phase 3 Trial of Concizumab in Hemophilia with Inhibitors. N. Engl. J. Med..

[42] Matino D., Palladino A., Taylor C.T., Hwang E., Raje S., Nayak S., McDonald R., Acharya S.S., Mahlangu J., Jiménez-Yuste V. (2025). Marstacimab prophylaxis in hemophilia A/B without inhibitors: Results from the phase 3 BASIS trial. Blood.

[43] Mullard A. (2024). FDA approves first anti-TFPI antibody for haemophilia A and B. Nat. Rev. Drug Discov..

[44] Magisetty J., Kondreddy V., Keshava S., Das K., Esmon C.T., Pendurthi U.R., Rao L.V.M. (2022). Selective inhibition of activated protein C anticoagulant activity protects against hemophilic arthropathy in mice. Blood.

[45] Jiang M., Yang F., Jiang Y., Cheng L., Han J., Yi J., Zuo B., Huang L., Ma Z., Li T. (2023). Safety and efficacy of an anti-human APC antibody for prophylaxis of congenital factor deficiencies in preclinical models. Blood.

[46] Prince R., Bologna L., Manetti M., Melchiorre D., Rosa I., Dewarrat N., Suardi S., Amini P., Fernández J.A., Burnier L. (2018). Targeting anticoagulant protein S to improve hemostasis in hemophilia. Blood.

[47] Barg A.A., Brutman-Barazani T., Avishai E., Budnik I., Cohen O., Dardik R., Levy-Mendelovich S., Livnat T., Kenet G. (2022). Anti-TFPI for hemostasis induction in patients with rare bleeding disorders, an ex vivo thrombin generation (TG) guided pilot study. Blood Cells Mol. Dis..

[48] Jalbert L.R., Rosen E.D., Moons L., Chan J.C., Carmeliet P., Collen D., Castellino F.J. (1998). Inactivation of the gene for anticoagulant protein C causes lethal perinatal consumptive coagulopathy in mice. J. Clin. Investig..

[49] Levy-Mendelovich S., Avishai E., Samelson-Jones B.J., Dardik R., Brutman-Barazani T., Nisgav Y., Livnat T., Kenet G. (2024). A Novel Murine Model Enabling rAAV8-PC Gene Therapy for Severe Protein C Deficiency. Int. J. Mol. Sci..

[50] Barg A.A., Livnat T., Kenet G. (2024). Factor XI deficiency: Phenotypic age-related considerations and clinical approach towards bleeding risk assessment. Blood.

[51] Cohen O., Santagata D., Ageno W. (2024). Novel horizons in anticoagulation: The emerging role of factor XI inhibitors across different settings. Haematologica.

[52] Fredenburgh J.C., Weitz J.I. (2023). News at XI: Moving beyond factor Xa inhibitors. J. Thromb. Haemost..

[53] Gailani D., Gruber A. (2024). Targeting factor XI and factor XIa to prevent thrombosis. Blood.

[54] Mannucci P.M. (2023). Hemophilia treatment innovation: 50 years of progress and more to come. J. Thromb. Haemost..

